# Coexistent Ankylosing Spondylitis and Ocular Toxocariasis in a Pediatric Patient Manifesting As Bilateral Panuveitis

**DOI:** 10.7759/cureus.82767

**Published:** 2025-04-22

**Authors:** Raymund V Tanchuling, Juan S Lopez, Roberto D Maliwat

**Affiliations:** 1 Ophthalmology, St. Luke's Medical Center, Quezon City, PHL; 2 Ophthalmology, Nueva Ecija Eye Center, Cabanatuan, PHL

**Keywords:** ankylosing spondylitis, bilateral uveitis, granulomatous panuveitis, ocular toxocariasis, pediatric uveitis

## Abstract

The coexistence of ankylosing spondylitis and ocular toxocariasis in the literature is rare and limited to a few case reports. Typically, such cases present as acute nongranulomatous anterior uveitis with *Toxocara* IgG seropositivity. A patient manifesting with findings of both ankylosing spondylitis and toxocariasis bilaterally has not been reported previously in the literature. We present a case of coexistent juvenile spondyloarthritis and ocular toxocariasis in a 16-year-old male presenting with generalized pustules, back pain, peripheral polyarthritis, and bilateral panuveitis. Both eyes displayed abnormalities in the anterior segments, including corectopia, seclusio pupillae, and occlusio pupillae. Posterior segment examination of the right eye showed vitritis, disc edema, and a retinochoroidal granuloma surrounded by infiltrates and perivascular sheathing. A B-scan of the left eye revealed vitritis and the presence of a hyperechoic band from the disc to the retinal periphery. *Toxocara* IgG and HLA-B27 were positive, and lumbosacral magnetic resonance imaging confirmed sacroiliitis. Treatment involved subtenon injections of triamcinolone and subcutaneous Etanercept injections, resulting in stabilization of visual acuity. This case highlights the rare co-occurrence of two diseases with overlapping symptoms and uncertain pathogenetic contributions from each to cause the observed manifestations. It supports studies proposing a connection between rheumatic disease and parasitosis.

## Introduction

Juvenile ankylosing spondylitis (JAS) is an inflammatory condition closely linked to HLA-B27 and most commonly presents with alternating acute anterior uveitis in roughly 90% of cases. Posterior segment involvement - such as vitritis or retinal vasculitis within the spectrum of panuveitis - is less common, occurring in approximately 10%-15% of patients [1]. While JAS typically emerges in early adolescence, it can manifest in children as young as five years old.

*Toxocara canis* is a ubiquitous parasite and one of the most common causes of zoonotic infection globally. The prevalence of infected puppies varies by region, with rates reported as 33% in London, 98% in Ohio, and 100% in Brisbane, Australia [2]. In humans, seroprevalence is estimated at around 19% worldwide, with some Southeast Asian studies reporting rates as high as 34% [3]. Transmission occurs through ingesting embryonated eggs from contaminated soil, water, raw produce, or undercooked meat. Ocular toxocariasis results when larval migration reaches the eye, typically producing a unilateral condition, with bilateral involvement being exceedingly rare, limited to a few case reports. Posterior segment involvement is most common, appearing in 50% of cases as a peripheral granuloma with or without vitreous haze, vitreous folds, retinal detachment, retinal vasculitis, macular edema, or exudation [4].

Although both JAS and ocular toxocariasis can independently lead to ocular inflammation, reports of their coexistence are scarce, and the sequence of onset remains uncertain. This case report details a patient with JAS who concurrently developed ocular toxocariasis manifesting as a bilateral panuveitis - a presentation not previously documented in an Asian Filipino patient.

## Case presentation

Patient history

A 16‐year‐old male presented with sequential blurring of vision, which initially manifested in the left eye (OS) before affecting the right eye (OD) two weeks later. A month later, the patient developed mild polyarthralgia with associated swelling of the hands, wrists, and ankles, with symptoms alternating between the extremities. In addition, he reported persistent low back pain, most pronounced nocturnally and ameliorated by activity, along with the onset of pustules distributed over the chest, back, and both upper and lower extremities, including the palms and soles, while sparing the mucosal surfaces. These systemic manifestations were accompanied by recurrent ocular complaints, notably pain, redness, blurred vision, photopsias, and floaters. His review of systems was otherwise unremarkable for constitutional, respiratory, gastrointestinal, or genitourinary symptoms. The patient’s past ocular history was noncontributory, with no antecedent trauma, surgical interventions, or previous similar episodes. Family history was negative for rheumatologic disorders. Social history was notable for prolonged exposure to domestic canines (three puppies and two adult dogs) without regular deworming, as well as a previous history of smoking and two heterosexual partnerships with consistent barrier protection. There was no history of consumption of uncooked meat or travel to areas deemed high risk for zoonotic infections.

Ocular examination

**Figure 1:**
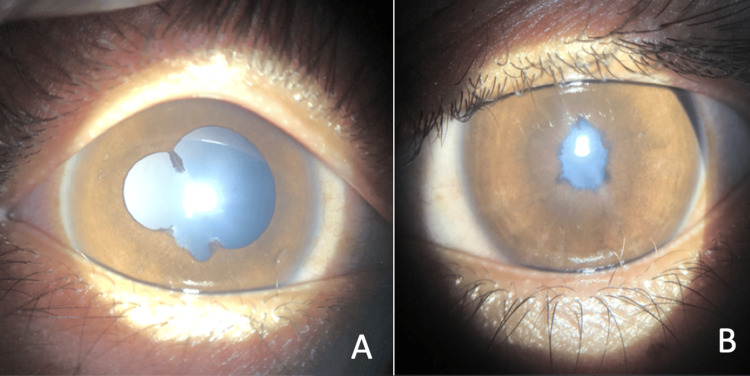
External exam findings of the right eye (A) with irregular, dilated, poorly reactive pupil, and posterior synechiae at 11, and 5-7 o’clock positions. Left eye (B) showing a small, nonreactive pupil, and occlusion membrane.

**Figure 2:**
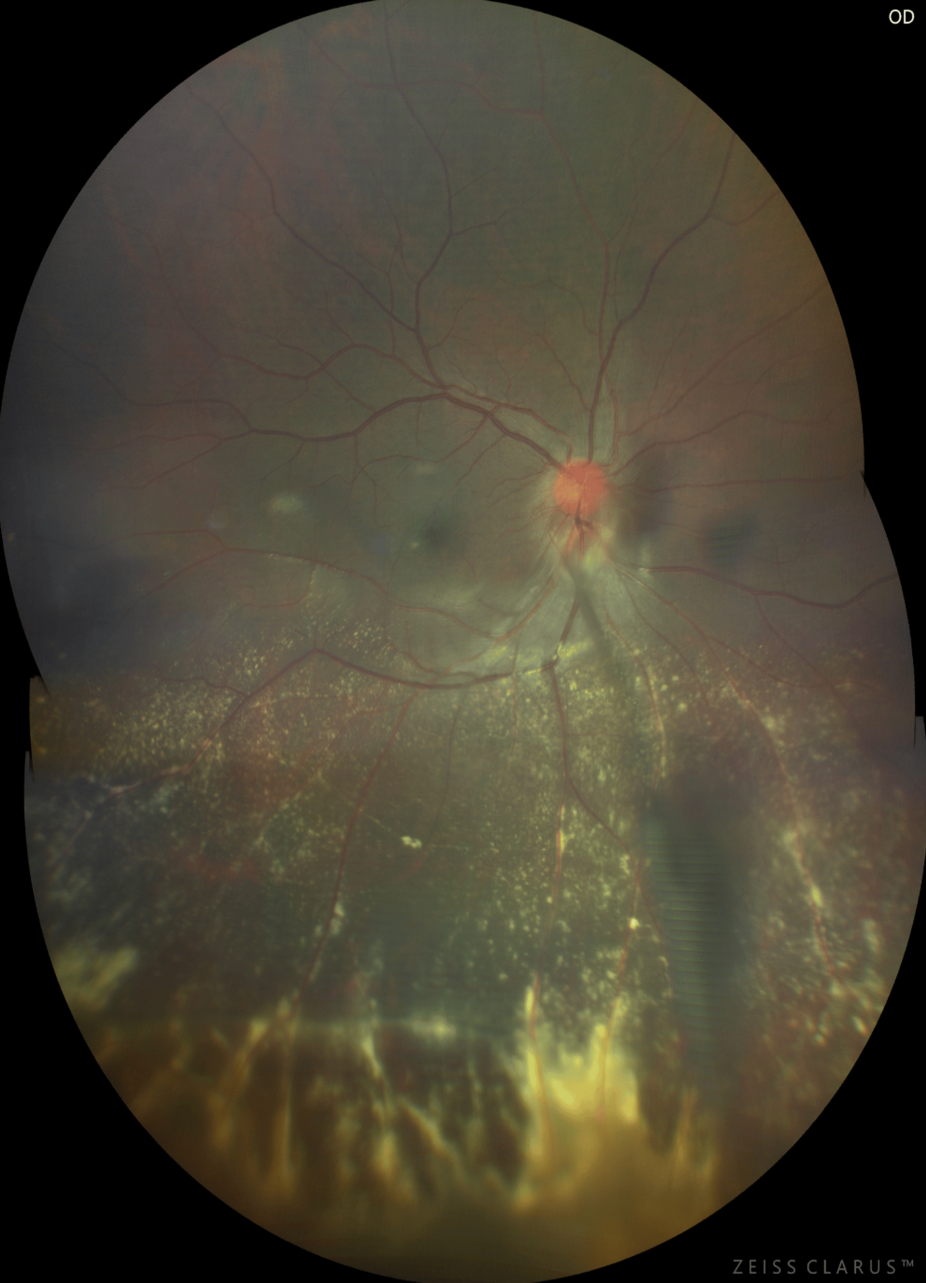
Ultrawide field fundus photograph of the right eye showing a hyperemic disc, retinal perivascular sheathing of both arteries and veins, confluent patches, retinal infiltrates, and exudates concentrated in the inferior retinal periphery, and a fibrovascular stalk from the disc to the noted area of intense inflammation. Indentation ophthalmoscopy revealed a peripheral granuloma surrounded by the confluence.

**Figure 3:**
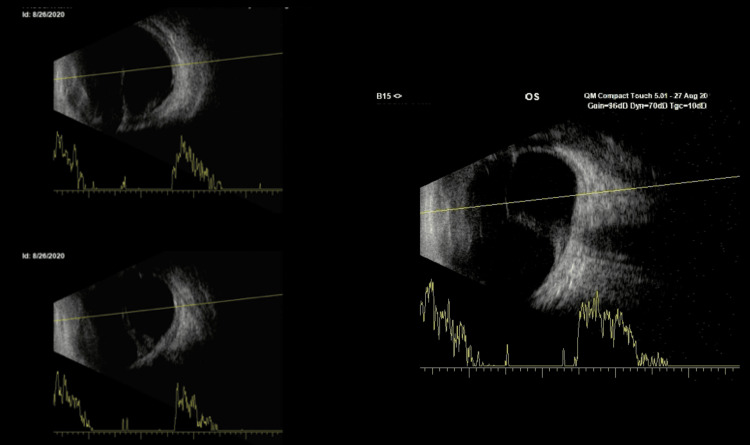
Axial scan of the left eye through closed eyelids showing a hyperechoic linear band with low to moderate amplitude echoes attaching to the optic disc and extending to the retrolental area and anterior retina.

The right eye (Figure 1A) exhibited a best-corrected visual acuity (BCVA) of 20/40, accompanied by an elevated intraocular pressure of 36 mmHg. Anterior segment examination of the right eye revealed a dilated, irregular, and poorly reactive pupil, with posterior synechiae at the 11, 5, and 7 o'clock positions. Fundoscopy (Figure 2) demonstrated a hyperemic optic disc, perivascular sheathing, confluent retinal infiltrates, and a fibrovascular stalk extending from the disc to the inferior peripheral retina. In contrast, the left eye (Figure 1B) had a BCVA of 20/400 and an intraocular pressure of 6 mmHg. The anterior segment findings in the left eye were notable for an occlusio membrane, and B-scan ultrasonography (Figure 3) revealed a hyperechoic band extending from the optic nerve to the anterior retina.

We noted multiple pustules over the chest, back, and arms, extending to the palms and soles without scaling. The rest of the systemic examination was unremarkable.

Imaging and laboratory results

**Figure 4:**
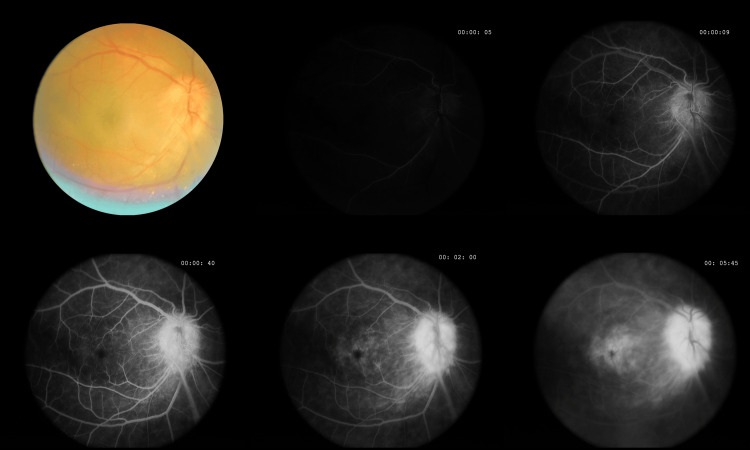
Fluorescein angiogram of the right eye showing early hyperfluorescence of the disc with intense leakage during transit, pooling of dye in the fovea in a petalloid pattern, and a hyperfluorescent band from the disc extending inferiorly correlating with the fibrovascular structure seen on color photo.

**Figure 5:**
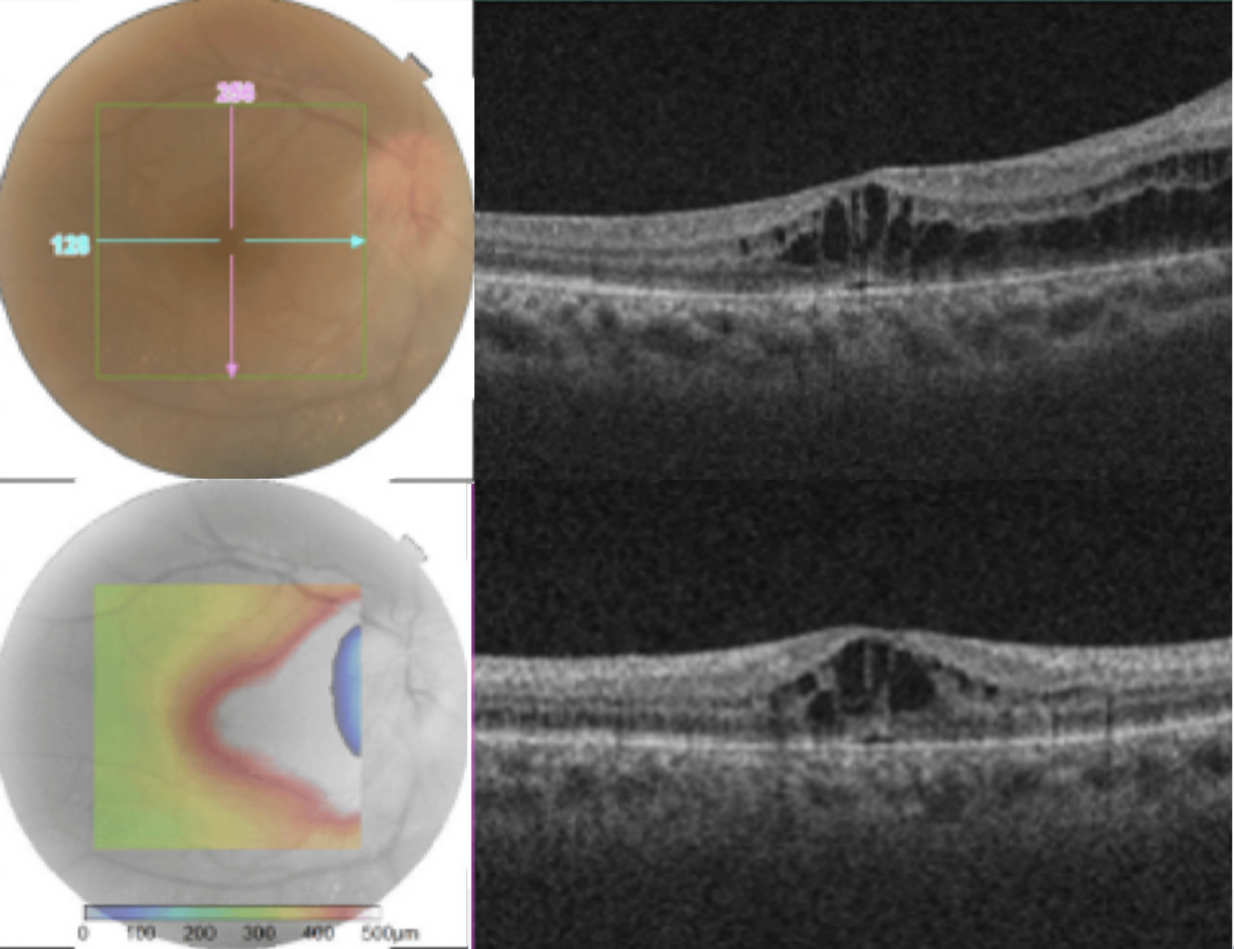
Macular OCT scan of the right eye showing cystoid changes in the fovea extending nasally to the disc, consistent with uveitic cystoid macular edema and optic disc edema

Fluorescein angiography of the right eye (Figure 4) demonstrated hyperfluorescent disc leakage, petalloid macular pooling, and a hyperfluorescent stalk extending inferiorly. Optical coherence tomography confirmed the presence of cystoid macular edema (CME) in the right eye (Figure 5).

**Figure 6:**
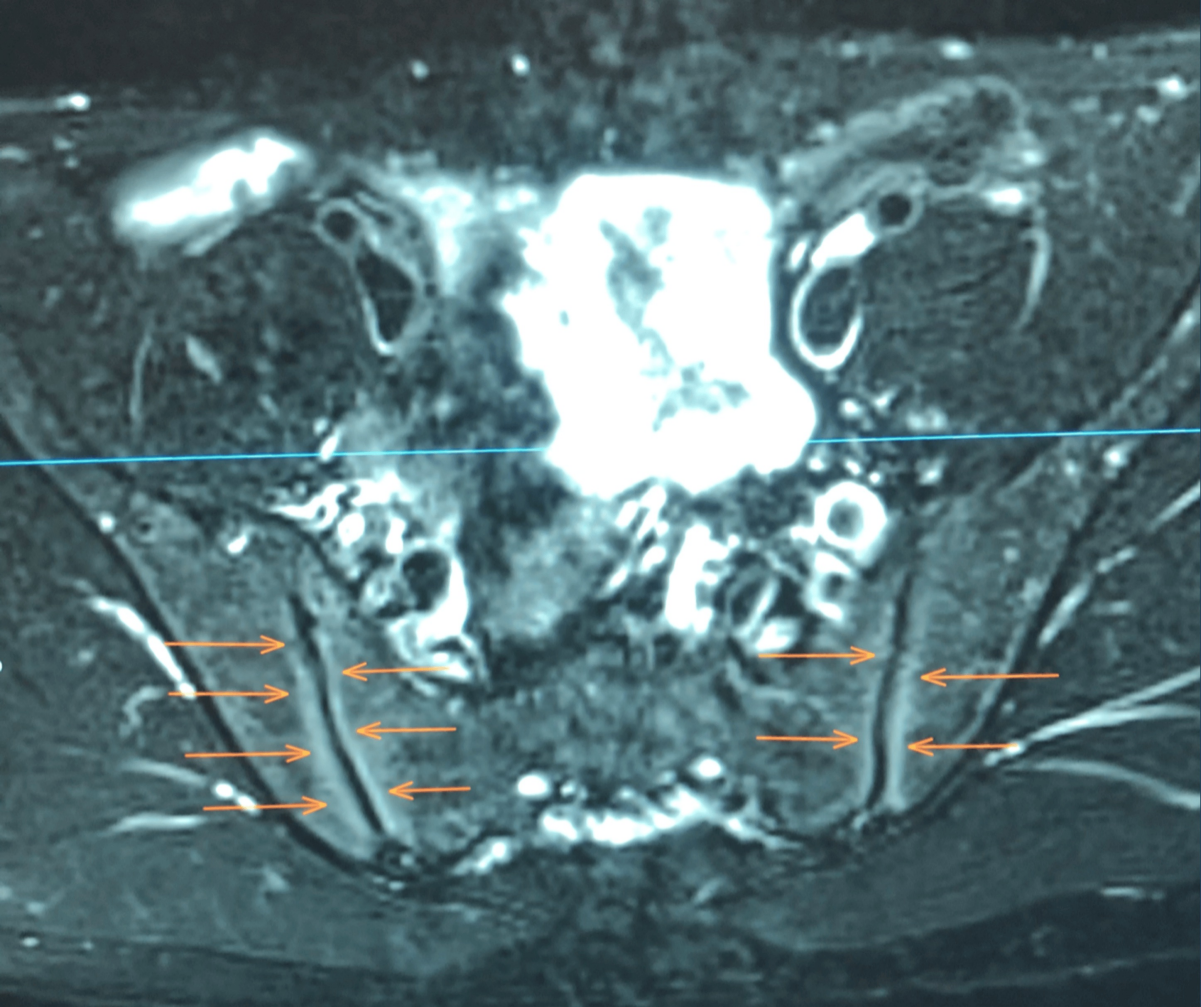
Definite sacroiliitis is shown as bone marrow hyperintensities (orange arrows) around both sacroiliac joints in this pelvic MRI.

Magnetic resonance imaging (MRI) of the sacroiliac joints (Figure 6) revealed bilateral sacroiliitis with bone marrow edema, findings consistent with JAS according to the criteria established by the International League of Associations for Rheumatology (ILAR) for juvenile idiopathic arthritis.

Serological testing was positive for both *Toxocara *IgG/IgM and HLA-B27. An extensive infectious and inflammatory work-up - including chest radiographs, Venereal Disease Research Laboratory/Rapid Plasma Reagin (RPR/VDRL), HIV screening, Mantoux test, liver enzymes (AST/ALT), two-dimensional echocardiography, rheumatoid factor, and antinuclear antibody testing - was unremarkable. The presence of elevated acute-phase reactants, including erythrocyte sedimentation rate (ESR), C-reactive protein (CRP), antistreptolysin-O (ASO), and platelets, further supported an underlying inflammatory process.

Management

A multimodal treatment approach was instituted to address both the systemic and ocular manifestations of the patient’s condition. Biologic therapy was initiated with etanercept at a dose of 50 mg subcutaneously once weekly to control the progression of ankylosing spondylitis (AS), mitigate inflammation, and prevent further increases in IOP. While studies mention that adalimumab is superior to etanercept for biologic-naive juvenile enthesitis-related arthritis (ERA) [5], our choice of anti-TNF inhibitor was mainly due to availability at the time of treatment.

Corticosteroid therapy was deemed essential for controlling both CME and ocular toxocariasis. A sub-Tenon’s triamcinolone acetonide injection of 40 mg was administered to achieve high intraocular drug concentrations, following a review suggesting systemic or periocular administration to help mitigate ocular inflammation in ocular toxocariasis [6].

To monitor disease activity, the Bath Ankylosing Spondylitis Disease Activity Index (BASDAI) [7] was utilized. The patient’s initial BASDAI score was calculated at 6, indicating suboptimal disease control and supporting the decision to initiate biologic therapy. Regular disease monitoring through BASDAI scoring allows for timely adjustments in medical management should disease activity persist or worsen.

Nonpharmacologic interventions were also considered to optimize the patient’s visual function and overall well-being. Sunglasses were recommended to reduce glare in the dilated right eye, and regular contrast sensitivity and visual acuity assessments were planned to monitor disease progression. Physical therapy and regular exercise were emphasized to improve joint and spinal mobility, potentially delaying the progression to ankylosis.

Given the systemic nature of JAS, comanagement with a rheumatologist was prioritized. The Dublin Uveitis Evaluation Tool (DUET) [8] was considered to assess the necessity of referral, while the Bath Ankylosing Spondylitis Toolkit guided disease management in both pediatric and adult patients.

Environmental modifications were also advised to minimize the risk of recurrent toxocariasis. Regular deworming of the patient’s pet dogs was recommended, along with strict hand hygiene and avoidance of soil contact. These preventive measures are crucial in reducing the risk of reinfection and further ocular complications.

The patient remains under close monitoring with regular follow-ups to assess treatment response and disease activity. Adjustments to therapy will be made based on clinical findings and objective disease activity scores.

## Discussion

This case highlights a rare co-occurrence of JAS and ocular toxocariasis, two conditions with overlapping ocular manifestations, making it challenging to delineate their contributions to the observed complications. The concurrent presentation of these diseases raises important considerations regarding their potential interaction and pathogenesis.

JAS was diagnosed based on the presence of arthritis, greater than three months of inflammatory back pain, sacroiliitis confirmed by MRI, HLA-B27 positivity, and symptomatic uveitis, as per the ILAR criteria for juvenile idiopathic arthritis. Ocular toxocariasis was diagnosed based on the identification of a peripheral chorioretinal granuloma with a vitreopapillary stalk and significant exposure to dogs, along with *Toxocara *IgG seropositivity [2], as outlined by Rubinsky-Elefant et al.

Anterior uveitis is the hallmark ocular feature of AS, but posterior or panuveitis also occurs in a minority of cases. Studies report posterior uveitis in 5%-46% and panuveitis in 7%-50% of AS patients, often manifesting with vitritis, retinal vasculitis, CME, or optic disc edema [1]. Such variability underscores the importance of imaging and detailed ocular examination in AS patients with atypical presentations. One study described a 36-year-old HLA-B27-positive man with longstanding AS who developed bilateral severe anterior chamber inflammation with marked vitritis [9]. Fluorescein angiography revealed extensive retinal vasculitis, optic disc leakage, and CME in both eyes, similar to our patient’s extensive posterior segment involvement and CME. However, our patient demonstrated bilateral granulomatous panuveitis, which is unusual for AS alone and suggests a more complex etiology. Additionally, unlike our case, the patient lacked evidence of granuloma or parasitic exposure.

Bilateral ocular toxocariasis, on the other hand, is extremely rare, with only a few documented case reports [10]. One such study reported a 56-year-old man who presented with hand-motion vision in the right eye and 20/30 vision in the left. He had a severe anterior chamber reaction OD and moderate reaction OS; funduscopic exam revealed a hemorrhagic granuloma with vascular sheathing in the left eye, while the right was obscured by dense vitreous opacities. ELISA confirmed *T. canis* IgG positivity in serum (1:38), aqueous (1:321 OD, 1:254 OS), and vitreous (1:679 OD). Combined oral albendazole, corticosteroids, and vitrectomy OD led to BCVA of 8/20 OD and 20/20 OS at six months, mirroring our patient’s bilateral granulomatous inflammation, serologic profile, and favorable response to antiparasitic plus anti-inflammatory therapy [11]. This patient, however, was older and had no autoimmune comorbidity, highlighting how our case is unique in its dual pathology.

Currently, there are no reports of coexistent AS and ocular toxocariasis in the literature, but there have been reports of the simultaneous presence of AS and parasitic infection. Jiménez-Balderas et al. found that 38% of AS patients with acute nongranulomatous anterior uveitis were seropositive for *T. canis* antibodies versus 7% without uveitis, suggesting chronic asymptomatic toxocariasis may contribute to uveitis pathogenesis [12]. Deveci and Kobak similarly reported posterior uveitis due to *Toxoplasma* in an AS patient on immunosuppressives, underscoring the need to consider parasitic etiologies in immunosuppressed AS patients to avoid delayed diagnosis and worse outcomes [13]. Moreover, a meta-analysis of over 8,900,000 subjects confirmed that prior infections increase AS risk (pooled OR 1.46, 95% CI 1.23-1.73; RR 1.35, 95% CI 1.12-1.63), supporting a broader role for infection-triggered immune dysregulation in AS development [14]. We believe that a similar mechanism may have been in play for our patient, where toxocariasis infection may have caused immune dysregulation, contributing to AS development.

Both AS and toxocariasis-related inflammation can lead to posterior synechiae, cataract formation, raised intraocular pressure, disc edema, and CME. In HLA-B27-associated uveitis, posterior synechiae occur in up to 90% of cases (13%-90%), cataracts in 7%-28%, ocular hypertension in 8%-20%, papillitis in 2%-18%, and CME in 6%-13% [1]. Ocular toxocariasis likewise has been implicated in cataract, vitreous opacities, CME, and optic disc edema, often requiring vitrectomy for membrane removal and visual rehabilitation [10]. In our patient, the convergence of these complications likely reflects additive or synergistic effects of both diseases.

This case contributes to the evolving understanding of how parasitic infections may serve as catalysts in autoimmune conditions like AS. While causality cannot be established from a single case, the patient's bilateral granulomatous panuveitis in the context of both HLA-B27 positivity and *Toxocara* seropositivity strengthens the argument for immune dysregulation as a shared pathological axis. Our findings underscore the clinical value of broadening diagnostic considerations in atypical forms of uveitis presentations and raise the possibility that early identification and treatment of latent infections could positively influence outcomes in autoimmune ocular disease. Future research with larger cohorts and mechanistic studies is essential to substantiate this potential immunologic interaction.

## Conclusions

This case underscores the critical need for a comprehensive diagnostic approach in pediatric panuveitis, particularly when rare and complex conditions coexist. The co-occurrence of JAS and ocular toxocariasis highlights the importance of considering both autoimmune and infectious causes in the differential diagnosis. While Occam’s razor advocates for diagnostic parsimony, Hickam’s dictum serves as a timely reminder that patients may present with multiple concurrent diseases, each contributing to the clinical picture. This case emphasizes the need for a multidisciplinary approach to effectively manage such complex cases. Furthermore, future research should delve deeper into the potential relationship between parasitic infections and autoimmune uveitis and investigate how these interactions influence ocular health, providing a clearer understanding of their pathophysiology and guiding more targeted treatment strategies.
